# Effects of grazing prohibition on *nirK*- and *nirS*-type denitrifier communities in salt marshes

**DOI:** 10.3389/fmicb.2023.1233352

**Published:** 2023-07-26

**Authors:** Niu Li, Jingrou Li, Ming Nie, Ming Wu, Jihua Wu

**Affiliations:** ^1^Ministry of Education Key Laboratory for Biodiversity Science and Ecological Engineering, Coastal Ecosystems Research Station of Yangtze River Estuary, School of Life Sciences, Institute of Biodiversity Science and Institute of Eco-Chongming, Fudan University, Shanghai, China; ^2^Wetland Ecosystem Research Station of Hangzhou Bay, Research Institute of Subtropical Forestry, Chinese Academy of Forestry, Hangzhou, Zhejiang, China

**Keywords:** denitrification, grazing prohibition, *nirK*- and *nirS*-type communities, salt marshes, soil management

## Abstract

**Introduction:**

Grazing prohibition is an effective management practice to restore salt marsh functioning. However, the effects of grazing exclusion on denitrifying microbial communities and their controlling factors in salt marshes remain unclear.

**Methods:**

In this study, we surveyed soil physicochemical properties and above- and below-ground biomass and using quantitative polymerase chain reaction and Illumina MiSeq high-throughput sequencing technology to determine the relative abundance, composition, and diversity of nitrite reductase *nirS*- and *nirK*-type denitrifying bacterial communities associated with grazing prohibition treatments and elevations.

**Results:**

The abundance of *nirS*-type denitrifiers increased with grazing prohibition time, whereas the abundance of *nirK*-type denitrifiers remained unaltered. Moreover, *nirS*-type denitrifiers were more abundant and diverse than *nirK*-type denitrifiers in all treatments. Grazing prohibition significantly altered the operational taxonomic unit richness, abundance-based coverage estimator, and Chao1 indices of the *nirS*-type denitrifying bacterial communities, whereas it only minimally affected the structure of the *nirK*-type denitrifying bacterial community.

**Discussion:**

The results imply that the *nirS* community, rather than *nirK*, should be the first candidate for use as an indicator in the process of salt marsh restoration after grazing prohibition. Substances of concern, total nitrogen, and salinity were the key environmental factors affecting the abundance and community composition of *nirS* and *nirK* denitrifiers. The findings of this study provide novel insights into the influence of the length of grazing prohibition and elevation on *nirS*- and *nirK*-type denitrifying bacterial community composition in salt marshes.

## 1. Introduction

Salt marshes are located in the transition zone between terrestrial and marine ecosystems and provide essential ecosystem services such as carbon storage (Costanza et al., 1997; Valiela and Cole, 2002; Zedler and Kercher, 2005). However, intensive human activities (e.g., livestock grazing) have had negative consequences on salt marshes, such as a reduction in aboveground vegetation productivity and coverage, invertebrate richness, and destruction of bird habitats, leading to soil erosion and disruption of the wetland structure and function (Eldridge and Delgado-Baquerizo, 2017). Consequently, to eliminate the negative effects of overgrazing on salt marshes, grazing prohibitions have been adopted by many governments worldwide to restore degraded salt marshes (Law et al., 2014). As the effective essential restoration means of salt marshes, previous research has focused primarily on the vegetation community (Esselink et al., 2002), soil properties (Lagendijk et al., 2017), carbon (C) (Spangler et al., 2021), and nitrogen (N) processes (Wang C. et al., 2019) that change under grazing prohibition. However, limited research has been conducted on the effects of grazing prohibition on soil microbes, specifically N-cycling functional microorganisms in salt marshes.

The soil nitrogen cycle is an essential component of the whole nitrogen cycle, and the N dynamics are driven by N-cycling microbes (Zhou et al., 2017; Song et al., 2019), which, to a certain extent, reflect the status of the ecosystem and are regarded as important biological indicators for ecosystem restoration (Bender et al., 2016).

Denitrification is the dominant natural pathway for the N cycling transformation (Hu et al., 2021) and plays an important role in nitrogen loss from salt marshes, removing more than 50% annual N from coastal soil and alleviating the high nitrogen loads of the salt marshes. Denitrification occurs under oxygen-limited conditions, and heterotrophic denitrifying bacteria produce gaseous nitrous oxide (N_2_O) or dinitrogen (N_2_) using organic matter as an electron donor and nitrate (NO_3_^−^) and nitrite (NO_2_^−^) as electron acceptors (Zumft, 1997; Liu et al., 2020). This denitrifying metabolic pathway involves many functional genes, e.g., nitrate reductases, atrial natriuretic peptide/factor, nitrite reductase (*nir*), non-ripening, and nitric oxide synthase (Chen et al., 2023). Among these, *nir* is widely used in the analysis of denitrifying bacterial community (Zheng et al., 2015). Notably, *nir* has two functionally equivalent forms with different structures: cytochrome c encoded by *nirS* and a Cu-containing enzyme encoded by *nirK* (Maeda et al., 2017). Sequence analysis of *nirS* and *nirK* provides a comprehensive measure of community diversity and has been effectively used to elucidate the community composition of denitrifying bacteria in various environmental samples (Francis et al., 2013). Therefore, *nirS* and *nirK* are often used as biomarkers to describe the abundance and diversity of denitrifying microorganisms (Abell et al., 2010).

As an effective management practice of degraded ecosystem, grazing prohibition affects soil physico-chemical properties, greenhouse gas emissions and the sucession of vegetation (Jing et al., 2014; Song et al., 2019; Kooch et al., 2020). Grazing prohibition often leads to higher aboveground litter return to the soil, and decrease in soil compaction due to absence of animal trampling, thus result in an increase in soil oxgen concertation. The effects of grazing prohibition on the composition and function of *nirS*- and *nirK* denitrfying bacterical communities in salt marshes remain relatively unexplored. Moreover, there is little information regarding how the duration of grazing prohibition influences *nirS*- and *nirK* denitrfying bacterical communities and and its drivering factors (Pan et al., 2016). Furthermore, environmental characteristics in salt marsh soil different from other systems, such as high salinity, lower redox potential, and the occurrence of periodic tidal inundation (Zhang et al., 2021; Li et al., 2022), so abundance and composition of *nirS*- and *nirK* denitrfying bacterical communities are likely influenced by elevations.

Chongming Island is the largest alluvial sand island in the world. It is located off the Shanghai Coast at the entrance of the Yangtze River, facing pressure from intensive urbanization and anthropogenic activity around the Yangtze River Delta (Wang et al., 2022; Zhang et al., 2022). Statistically, Chongming Island is facing a high loss of wetlands and a decline in ecosystem function owing to unreasonable wetland use (Zhan and Xie, 2022). The direct conversion of wetlands into grazing land is one of the main anthropogenic impacts on wetlands, resulting in a reduction in vegetation communities and invertebrate diversity, alteration of carbon resources, reduction in birds’ food resources, and destruction of bird habitats (Yang Z. et al., 2017). Recently, the Chinese government has promoted the construction of eco-islands and proposed Chongming Island as a world-class eco-island in China (Peng et al., 2021). Therefore, grazing prohibition was implemented to remediate and restore Chongming Island wetlands. This restoration effort protected and gradually restored Chongming Island to a certain extent. During the restoration process, the denitrification ability of the salt marsh improved (Li et al., 2022). However, little is known about the changes in *nirS*- and *nirK*-denitrifying microorganisms involved in this process associated with grazing prohibition time at different elevations.

To determine how the soil *nirS*- and *nirK*-type denitrifiers respond to grazing prohibition in salt marshes, we hypothesized that soil physicochemical properties and *nirS*- and *nirK*-type denitrifiers respond differently to grazing prohibition time in the high and middle marshes. The purpose of this study was (a) to identify the difference between *nirS*-and- *nirK*-denitrifying bacterial communities along with grazing prohibition time in high and middle marshes, and (b) to evaluate the underlying mechanisms of the influence of grazing prohibition on *nirS*- and *nirK*-type denitrifiers.

## 2. Materials and methods

### 2.1. Description of study area

**Figure 1:**
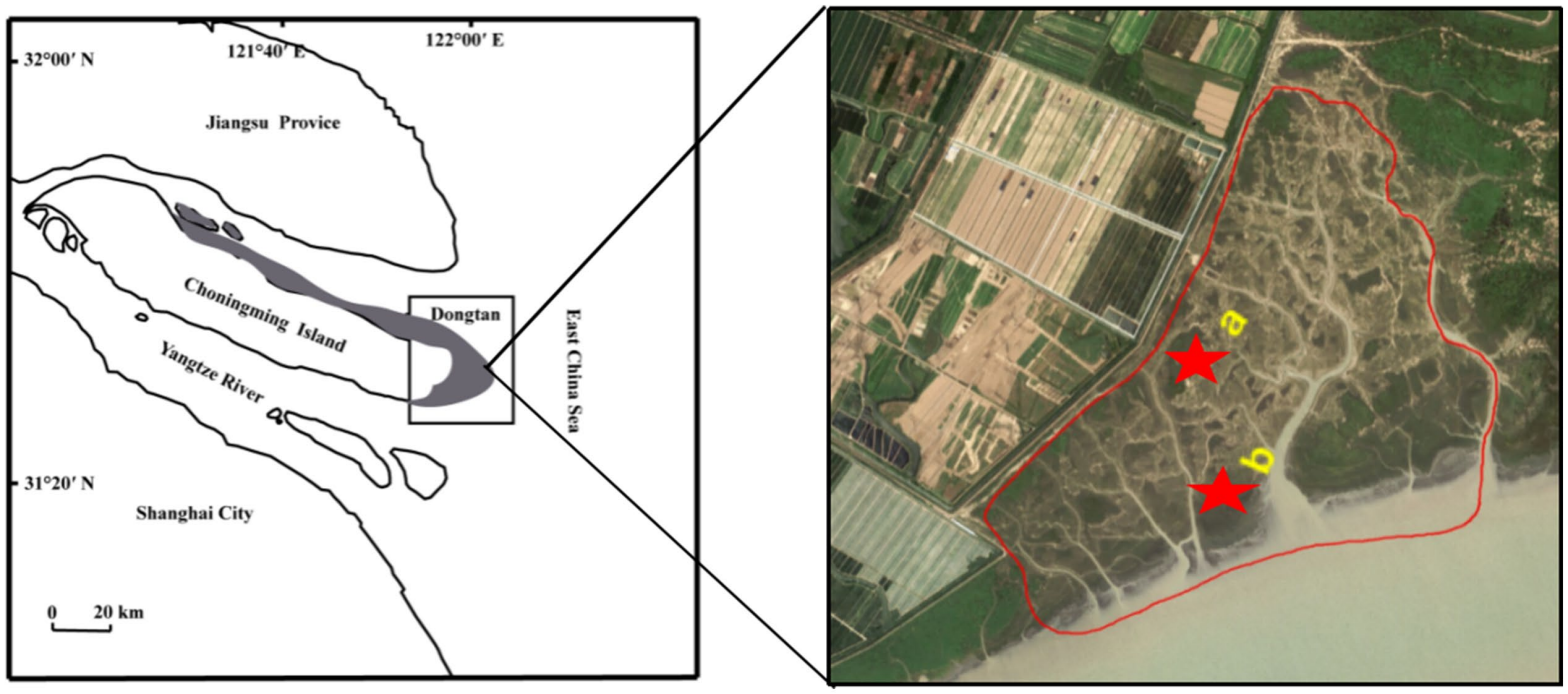
Location of the grazing prohibition sites in Dongtan salt marsh from Chongming Island, China. The red circle in the picture represents the entire grazing prohibition area. (a) high marsh; (b) middle marsh.

The research area of approximately 4,000 km^2^ is located on Chongming Island, China (31°69′N, 121°65′E) (Figure 1). This region has a subtropical monsoon climate with an annual precipitation and temperature of 1,145 mm and 15.3°C, respectively (Yang Z. et al., 2017). Tidal fluctuations in the vicinity of the Dongtan salt marsh were regular and semidiurnal. The ebb and flood tides are two distinct diurnal tidal periods. The average elevations of the high and middle marshes were 380 and 330 cm above sea level, respectively, resulting in average monthly inundation frequencies of 17 and 39, respectively. The vegetation is dominated by *Phragmites australis*, *Carex scabrifolia, Imperata cylindrica, and Scirpus mariqueter* (Li et al., 2022). The grazing history in this region can be traced back to 1949, before the establishment of the People’s Republic of China (Tang et al., 2020). Cattle grazing disturbs bird habitats, changes plant community composition and structure, and damages the environment in the area (Yang Z. et al., 2017). Since 2011, the local government has implemented grazing prohibition policies during different periods (Figure 1).

### 2.2. Experimental design and sampling

High and middle marshes with different durations of grazing prohibition were selected in August 2018. Grazing has been prohibited at these sites since 2017, 2014, and 2011, corresponding to grazing prohibitions of 1 (GP1), 4 (GP4), and 7 years (GP7), respectively. Before the prohibition, these sites were heavily grazed, with one cattle grazing per hectare. Detailed information on all the sites is presented in Table 1.

**Table 1:** Basic information of site characteristics.

Marsh zone	Grazing prohibition treatment	Latitude (N)	Longitude (E)	Community type
High marsh	GP1	31°28′31”	121°56′35”	*Phragmites australis, Carex scabrifolia, Imperata cylindrica, Scirpus mariqueter*
	GP4	31°28′33”	121°56′36”	*Phragmites australis, Carex scabrifolia,mperata cylindrica*
	GP7	31°28′52”	121°56′56”	*Phragmites australis*
Middle marsh	GP1	31°28′30”	121°56′51”	*Phragmites australis, Carex scabrifolia, Imperata cylindrica, Scirpus mariqueter, Scirpus validus*
	GP4	31°28′14”	121°57′49”	*Phragmites australis, Carex scabrifolia, Imperata cylindrica*
	GP7	31°28′31”	121°57′16”	*Phragmites australis*

GP1, grazing prohibition for 1 year; GP4, grazing prohibition for 4 years; GP7, grazing prohibition for 7 years.

Five replicate plots (15 m × 15 m) were randomly established within the grazing prohibition sites at a distance of 80–100 m between each other. Soil samples were randomly collected from nine points (3.8 cm diameter, 15 cm depth) within each plot and mixed as one sample. After the roots, litter, and debris were removed, the collected soil was passed through a 2 mm mesh and then divided into two subsamples. One subsample was immediately stored at −80°C for DNA analysis, and the other was airdried for physicochemical analysis. Moreover, in each plot, we randomly selected three subplots (25 × 25 cm) to measure the cover, species number, aboveground biomass, and root biomass. Roots were collected from the same quadrats using a polyvinyl chloride tube (15 cm diameter, 20 cm depth) (Wei et al., 2020). The roots were immediately washed in clean water to remove all soil, and then both the aboveground living plant tissues and roots were oven dried at 80°C to a constant weight.

### 2.3. Soil physicochemical analysis

Redox potential (Eh) and soil temperature were measured using multiple digital meters (IQ Scientific Instruments, CA, United States) (Rostaminia et al., 2011). The soil bulk density was determined using the cutting ring method (Fang et al., 2020). Moisture content was determined by oven-drying fresh soil at 105°C for 24 h (Herold et al., 2018). Soil pH and salinity were measured using a conductivity meter (soil:water = 1:2.5; SevenExcellenceS479-uMix, Mettler-Toledo, Switzerland) (Li et al., 2009). To determine the total nitrogen (TN), the soil was passed through 100 mensh sieve and analyzed using an N/C Soil Analyzer (Flash EA 11121 Series; ThermoFinnigan, Milan, Italy) (Wang C. et al., 2019). Ammonium (NH_4_^+^) and nitrate (NO_3_^−^) were measured using a continuous flow analyzer (SAN^++^, Skalar, Netherlands) after extraction in a 2 M KCl solution at a 1:5 w:v ratio (Yang Y. et al., 2017). Soil organic carbon (SOC) was determined as weight loss on ignition at 550°C for 5 hours (Wang et al., 2020).

### 2.4. DNA extraction and quantitative polymerase chain reaction

Total genomic DNA was extracted from the mixed frozen soil (~ 0.25 g) using a PowerSoil DNA Isolation Kit (MOBIO, Qiagen, Germany), according to the manufacturer’s instructions. The DNA concentration and purity were assessed using a NanoDrop 2000 ultraviolet (UV)-vis spectrophotometer (ThermoFisher Scientific) and validated using 0.8% agarose gel electrophoresis spectrophotometer, respectively.

### 2.5. Quantitative polymerase chain reaction

Quantitative polymerase chain reaction (qPCR) was performed in triplicate to study the abundances of *nirS* and *nirK* using an Applied Biosystems (ABI) 7,500 Detection system (Life Technologies, United States) and the SYBR Green method. The primer pairs *nirS*-cd3aF (GTSAACGTSAAGGARACSGG) and *nirS*-R3cd (GASTTCGGRTGSGTCTTGA), as well as *nirK*-F1aCu (ATCATGGTSCTGCCGCG) and *nirK*-R3Cu (GCCTCGATCAGRTTGTGGTT), were used to quantify the abundance of *nirS* and *nirK*, respectively (Hallin and Lindgren, 1999; Throbäck et al., 2004). The qPCR reaction mixture (20 μL) contained 2 × SYBR Color qPCR Master Mix (16.4 μL), 0.5 μM of each primer, and 2 μL of DNA template. The PCR reaction conditions were as follows: 5 min of initial denaturation at 95°C, 40 cycles of denaturation for 15 s at 95°C, 30 s of annealing at 60°C, and 40 s of elongation at 72°C. The amplification efficiencies of *nirS* and *nirK* were > 90%, and their correlation coefficients were 0.994 and 0.997, respectively.

### 2.6. Illumina MiSeq sequencing and data analysis

The primers *nirS*-cd3aF, *nirS*-R3cd, *nirK*-F1aCu, and *nirK*-R3Cu have also been used to analyze denitrifying communities (Murdock and Juniper, 2017). Functional genes were first subjected to PCR amplification using an ABI GeneAmp 9,700 PCR system (Applied Biosystems, Foster City, CA, United States). The reaction solution was of 20 μL, containing 4 μL of 5 × FastPfu Buffer, 2 μL of deoxynucleoside triphosphates (2.5 mM), 0.8 μL of each primer (5 μM), 0.4 μL of FastPfu polymerase, 0.2 μL of bovine serum albumin, and 10 ng of template DNA. The mixture was then adjusted to the required volume with Milli-Q water. Amplification conditions were as follows: 95°C for 3 min, 37 cycles of 95°C for 30 s, 55°C for 30 s, and 72°C for 45 s, with a final extension at 72°C for 10 min. High-throughput sequencing (Illumina MiSeq) was performed by Biomarker Technologies Co. Ltd. (Beijing, China) using an Illumina MiSeq platform. Raw *nirS* and *nirK* gene sequencing reads were quality-filtered using Trimmomatic and FLASH. Operational taxonomic units (OTUs) were determined using USEARCH software, and sequences with 97% similarity were assigned to the same OTU (Chen et al., 2020). Alpha diversity indices (Chao 1, Shannon, and Simpson) were obtained using MOTHUR. Beta diversity was generated to assess the differences in community composition between grazing prohibition treatments using principal coordinate analysis at the OTU level based on the Bray–Curtis metric on the “quantitative insights into microbial ecology” platform (Yang et al., 2021).

### 2.7. Statistical analysis

Two-way analysis of variance (ANOVA) was performed to evaluate the effects of grazing prohibition time and marsh zone on soil properties, *nirK*- and *nirS*-type denitrifier gene abundances, and diversity indices. ANOVA was performed using SPSS Statistics (version 19.0; SPSS Inc., Chicago, IL, United States). The significance of the differences was calculated using Tukey’s honestly significant difference test (*p* < 0.05). Values were expressed as the mean (*n* = 5) ± standard error (SE). The non-metric multidimensional scaling (NMDS) based on Bray–Curtis distance of the *nirK*- and *nirS*-type denitrifiers between different grazing prohibition times were conducted using the “Vegan” package, and the “ggplot2” package in R software was used to visualize data. To identify relationships between environmental variables and the abundances and communities of *nirS*- and *nirK*-denitrifiers, Spearman correlation analyses, Mantel’s test and redundancy analyses (RDA) were performed using R Studio software version 3.5.1 (RStudio, Inc., Boston, MA, United States). Furthermore, hierarchical partitioning method was further use to identify the environmental variables contributing to the total soil denitrifying bacterial community by grouping them according to a hierarchical structure.

## 3. Results

### 3.1. Plant and soil properties

**Figure 2:**
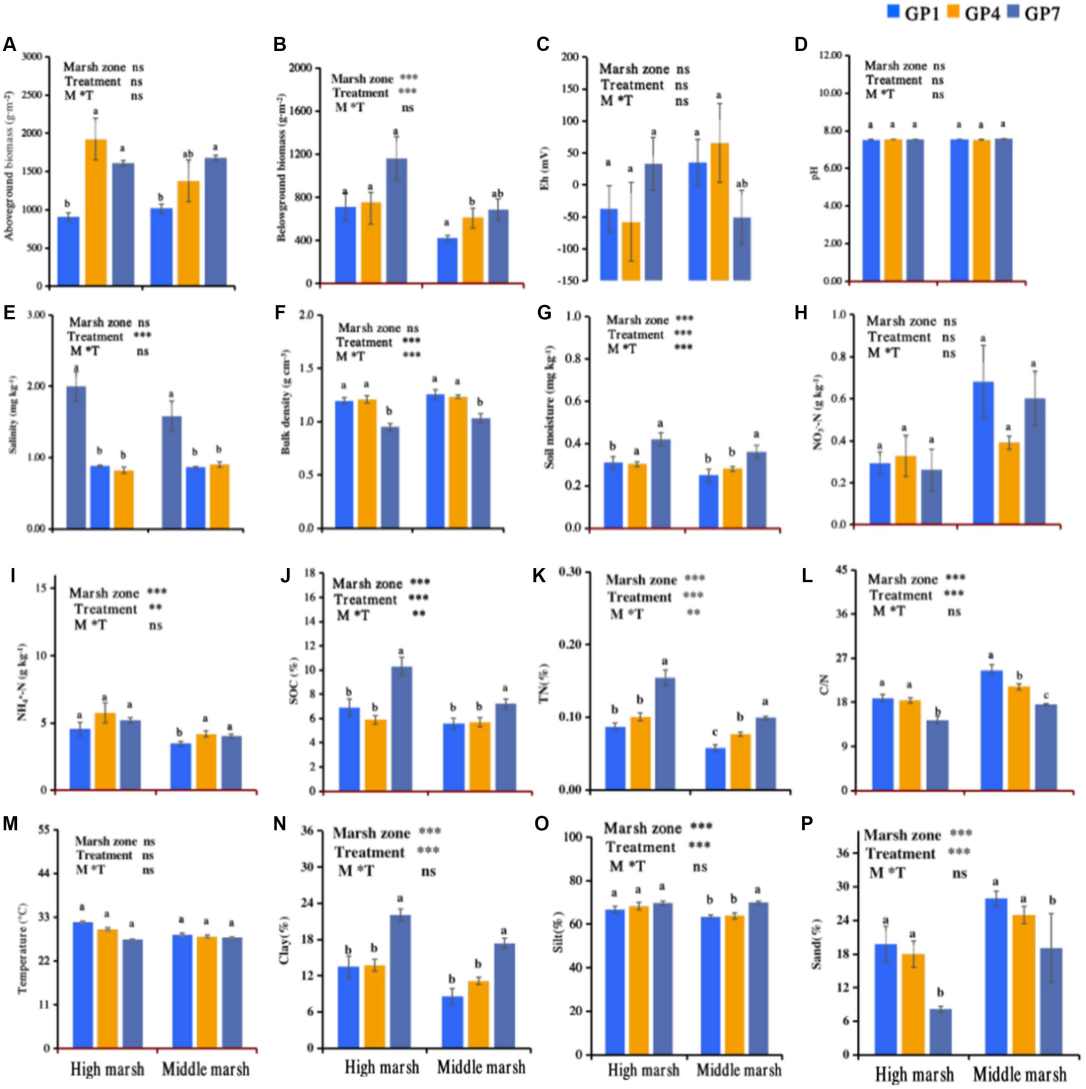
Effects of grazing prohibition on plant and soil properties. Mean ± standard error, *n* = 5; GP1: grazing prohibition for one year; GP4: grazing prohibition for four years; GP7: grazing prohibition for seven years. Different letters indicate significant differences (*p* < 0.05) in plant and soil properties between grazing prohibition times. The ns and asterisk symbols ** and *** indicate non-significant, and significant difference at *p* < 0.01 and *p* < 0.001, respectively. **(A)** Aboveground biomass; **(B)** Belowground biomass; **(C)** Eh; **(D)** pH; **(E)** Salinity; **(F)** Bulk density; **(G)** Moisture; **(H)** NO3--N; **(I)** NH4+-N; **(J)** SOC; **(K)** TN; **(L)** C/N; **(M)** Temperature; **(N)** Clay; **(O)** Silt; **(P)** Sand.

Grazing prohibition in the salt marsh was beneficial to the recovery of the plant community, which showed higher above-and belowground biomass along with grazing prohibition time (Figures 2A,B). The highest soil moisture, SOC, TN, clay, and silt were observed in GP7 treatment, followed by GP4 and GP1 in a decreasing order (Figures 2G,J,K,N,O). However, soil salinity, bulk density, C/N, and sand content decreased with grazing prohibition time (Figures 2E,F,L,P). Moreover, the pH values and the content of NH_4_^+^, NO_3_^−^, Eh and temperature did not change markedly with restoration time (Figures 2C,D,H,I,M).

### 3.2. Abundances of *nirS* and *nirK*

**Figure 3:**
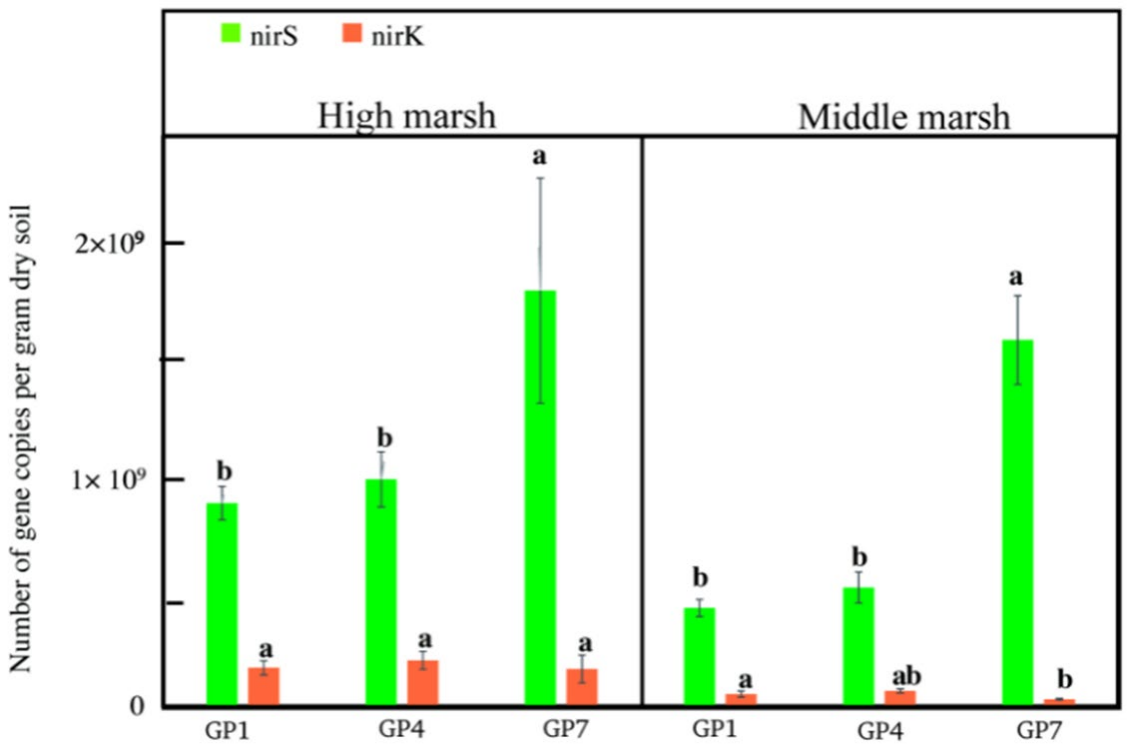
Abundance of *nirK* and *nirS* sequences from soil in the different treatments. GP1: grazing prohibition for one year; GP4: grazing prohibition for four years; GP7: grazing prohibition for seven years. Error bars indicate standard errors and different letters indicate significant differences (*p* < 0.05) between different grazing prohibition treatments.

Abundance of *nirS* ranged from 8.9 × 10^8^ to 1.82 × 10^9^ and 4.31 × 10^8^ to 1.61 × 10^9^ copies g^−1^dry soil in the high and middle marshes, respectively. The *nirK* abundance ranged from 1.64 × 10^8^ to 2.02 × 10^8^ copies g^−1^dry soil in the high marsh and from 3.11 × 10^7^ to 6.76 × 10^7^ copies g^−1^dry soil in the middle marsh (Figure 3). Clearly, the *nirS* gene copies were more abundant than *nirK* gene copies in the salt marsh soil, and the marsh zone had effect on *nirK* but no *nirS* gene copies during this experiment (Supplementary Table S1). The *nirS* gene copy numbers increased with grazing prohibition time and achieved a maximum at the GP7 treatment, with *nirS* gene copies 51% and 45% higher in high marsh and 73% and 67% higher in middle marsh than those of GP1 and GP4, respectively (Figure 3; Supplementary Table S1). However, the grazing prohibition time had no effect on *nirK* gene copy number (Figure 3).

### 3.3. Alpha diversity of *nirS*- and *nirK*-type denitrifying microorganisms

Grazing prohibition in the middle marsh significantly affected the richness of *nirK*-type denitrifiers (Supplementary Table S2). The OTU richness, ACE, and Chao1 indices of GP4 and GP7 were significantly higher than those of GP1, whereas there was no obvious change between the grazing prohibition periods in the high marsh (Table 2). For the diversity indices, neither the grazing prohibition treatment nor marsh zone affected the Simpson and Shannon indices of *nirK*-type denitrifiers (Table 2; Supplementary Table S1). The richness of *nirS*-type denitrifiers was also influenced by grazing prohibition time. OTU richness, ACE, and Chao1 indices increased with grazing prohibition time. However, there were no significant differences in the Simposon and Shannon indices between grazing prohibition times in the salt marsh soil (Table 2; Supplementary Table S1). Moreover, the richness indices of *nirS*-type denitrifiers were much higher than those of *nirK*-type denitrifiers, and no significant difference was detected in the diversity indices between *nirS*-type and *nirK*-type denitrifiers.

**Table 2:** The comparison of the alpha diversity of *nirK* and *nirS* gene clones in different treatments.

Marsh zones	Genes	Treatments	OTU richness	ACE	Chao1	Simpson	Shannon
High marsh	*nirK*	GP1	511 ± 16a	542.73 ± 13.43a	557.45 ± 11.35a	0.023 ± 0.001b	4.271 ± 0.029b
		GP4	514 ± 28a	538.31 ± 28.79a	547.77 ± 30.34a	0.027 ± 0.003b	4.591 ± 0.073a
		GP7	443 ± 40a	471.69 ± 39.78a	477.58 ± 37.34a	0.044 ± 0.006a	4.082 ± 0.016a
	*nirS*	GP1	604 ± 14b	638.69 ± 15.26b	646.58 ± 15.18b	0.026 ± 0.002a	4.551 ± 0.049a
		GP4	681 ± 9a	710.99 ± 10.23a	718.04 ± 10.55a	0.022 ± 0.001a	4.672 ± 0.050a
		GP7	683 ± 10a	715.11 ± 9.11a	719.68 ± 11.76a	0.023 ± 0.001a	4.637 ± 0.043a
Middle marsh	*nirK*	GP1	308 ± 26b	330.56 ± 27.76b	337.11 ± 31.58b	0.047 ± 0.016a	4.046 ± 0.023a
		GP4	468 ± 49a	496.69 ± 27.67a	496.69 ± 52.53a	0.030 ± 0.001a	4.402 ± 0.04a
		GP7	473 ± 19a	501.56 ± 20.07a	501.56 ± 20.07a	0.041 ± 0.005a	4.252 ± 0.013a
	*nirS*	GP1	682 ± 1b	698.78 ± 1.37b	701.85 ± 3.49b	0.020 ± 0.001a	4.868 ± 0.023a
		GP4	694 ± 7b	711.93 ± 6.30b	718.65 ± 4.46b	0.018 ± 0.001a	4.954 ± 0.041a
		GP7	711 ± 7a	735.04 ± 7.18a	741.11 ± 9.02a	0.017 ± 0.001a	4.899 ± 0.049a

ACE, abundance-based coverage estimator; Chao1, Chao’s species richness estimator; Shannon, Shannon-Weiner index; Simpson, Simpson’s diversity index. Species level, 97% similarity threshold used to define operational taxonomic units (OTUs); GP1, grazing prohibition for 1 year; GP4, grazing prohibition for 4 years; GP1, grazing prohibition for 7 years. Different letters indicate significant differences (*p* < 0.05) in the alpha diversity of *nirK* and *nirS* between various grazing prohibition times.

### 3.4. Beta diversity of *nirS*- and *nirK*-type denitrifying microorganisms

**Figure 4:**
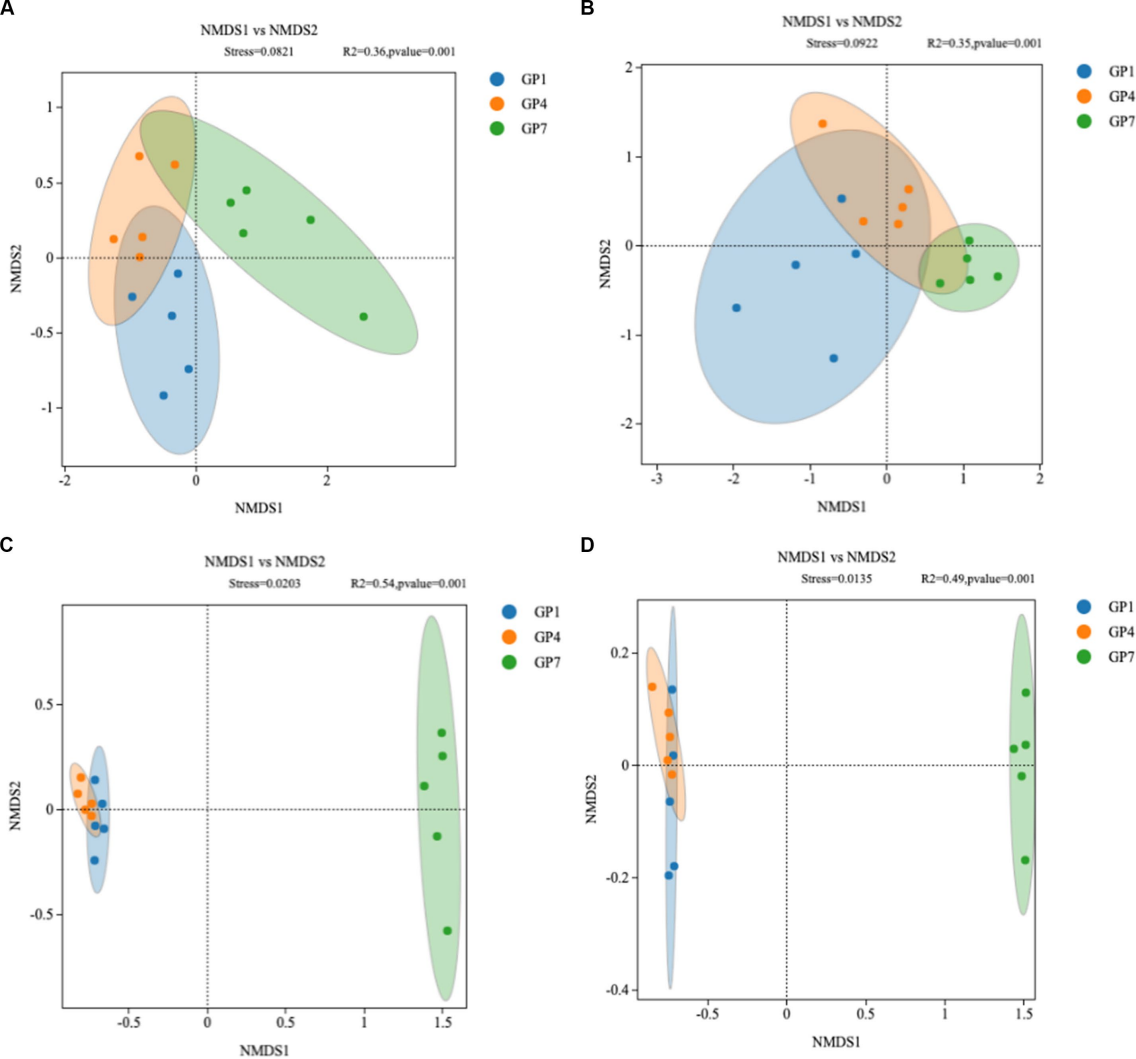
Non-metric multidimensional scaling analysis of *nirK*-type denitrifying communities in high marsh **(A)** and middle marsh **(B)** and *nirS*-type denitrifying communities in high marsh **(C)** and middle marsh **(D)** under different treatments. Treatments: GP1: grazing prohibition for one year; GP4: grazing prohibition for four years; GP7: grazing prohibition for seven years.

NMDS analysis showed no significant difference in the community composition of soil *nirK* denitrifying microorganisms between each treatment in the high marsh [Analysis of similarities (ANOSIM) *R* = 0.082, *p* = 0.36] and middle marsh (ANOSIM *R* = 0.092, *p* = 0.35) (Figures 4A,B). However, in the *nirS*-type denitrifying community, GP7 was separated from the other two treatments, but such shifts were not statistically significant between GP1 and GP4 in the high marsh (ANOSIM *R* = 0.020, *p* = 0.54) or middle marsh (ANOSIM *R* = 0.014, *p* = 0.49) (Figures 4C,D). These results indicated that grazing prohibition could affect the community composition of denitrifying microorganisms and that the *nirS*-type denitrifying community was more responsive to grazing prohibition.

**Figure 5:**
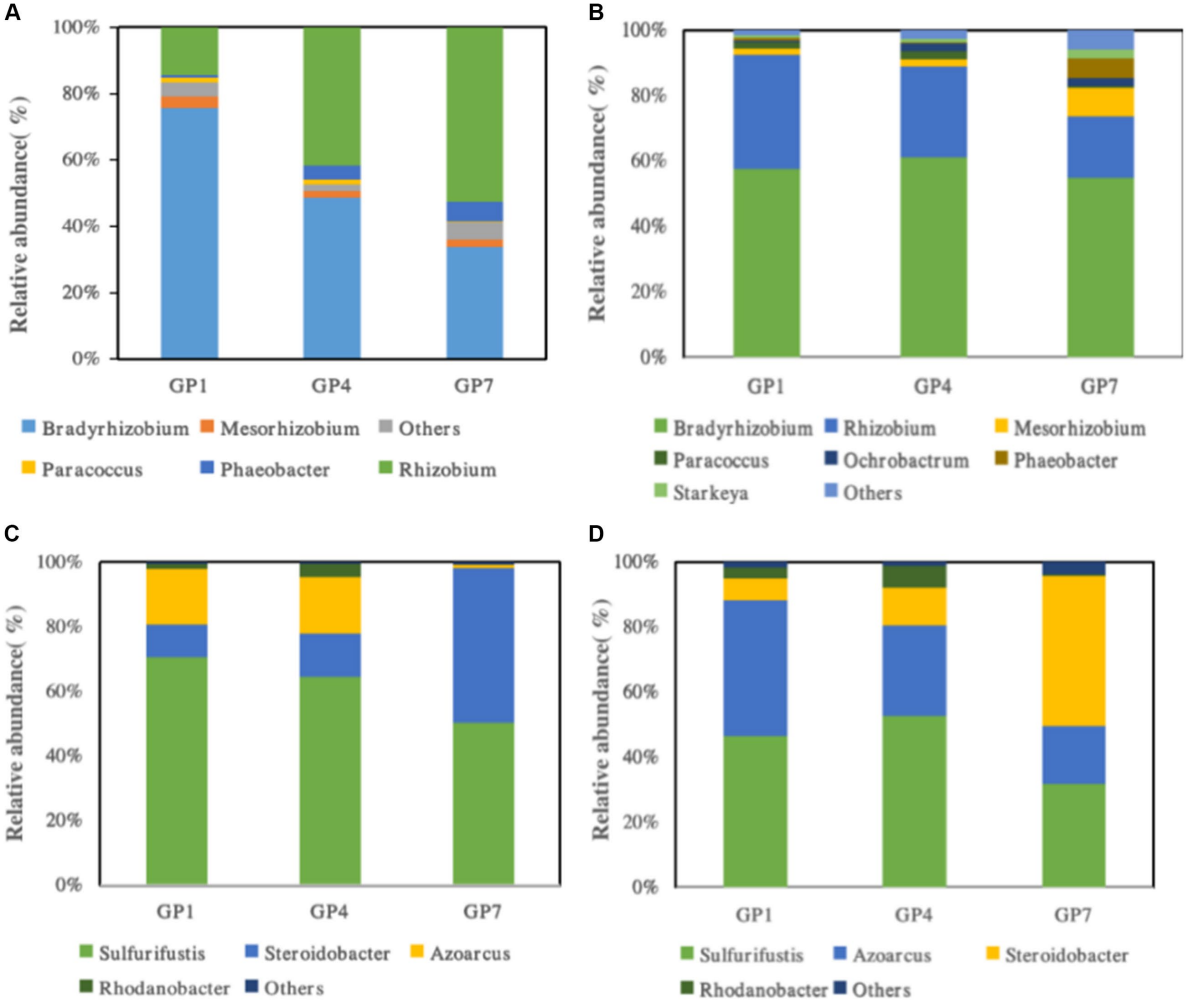
The community compositions of *nirK*-type denitrifying communities in high marsh **(A)** and middle marsh **(B)** and *nirS*-type denitrifying communities in high marsh **(C)** and middle marsh **(D)** under different treatments.

The relative community abundances of *nirS*- and *nirK*-type denitrifiers at the genus level in the high and middle marshes are shown in Figure 5. The proportion of groups classified as “Others” in the total sequence was less than 0.1%. For *nirK*-type denitrifier communities, it was shown that *Bradyrhizobium* (43.3%–72.6%, 21.2%–72.9%), *Rhizobium* (13.9%–37.5%, 33.1%–63.7%), *Mesorhizobium* (1.5%–3.1%, 2.6%–3.4%) in high and middle marsh were the predominant groups in GP1, GP4, and GP7 soils, respectively (Figures 5A,B). The relative abundance of *Bradyrhizobium* and *Rhizobium* was significantly affected by grazing prohibition. In the *nirS*-type denitrifying communities, we observed that the predominant genus was *Thiobacillus*, accounting for 3.6%–9.8% of the total *nirS* sequences (Figure 5B). In the *nirS*-type denitrifying community, we found that the predominant genera were *Sulfurifustis* (39.3%–62.7%, 18.2%–38.9%), *Steroidobacter* (3.3%–37.4%, 5.5%–26.3%), *Azoarcus* (6.4%–15.4%, 10.2%–35.0%) in high and middle marsh, respectively (Figures 5C,D). The relative abundances of *Sulfurifustis* and *Azoarcus* evidently increased, but *Steroidobacter* decreased with grazing prohibition time.

### 3.5. Possible drivers of denitrifying bacterial community composition

**Figure 6:**
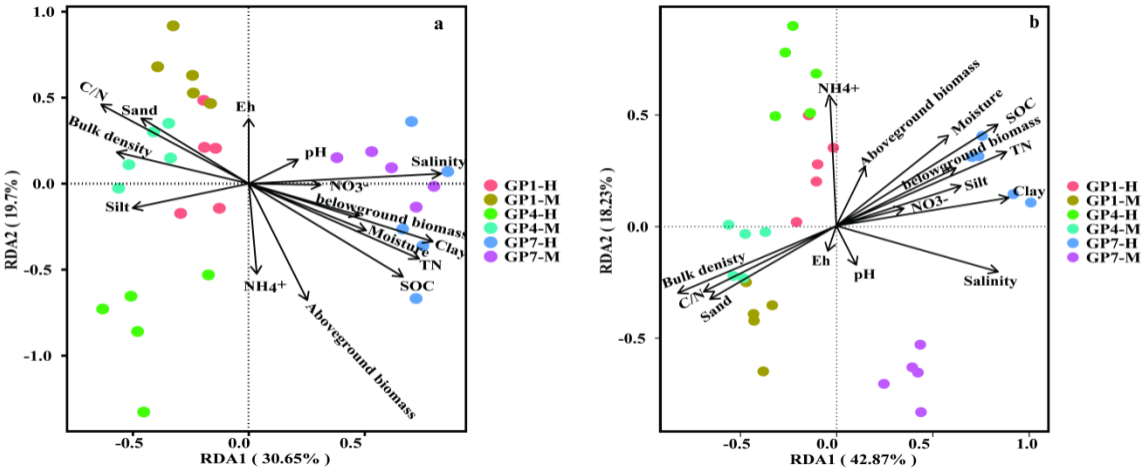
Redundancy analysis (RDA) of denitrifying communities and soil properties from different treatments. **(A)** *nirK*, **(B)** *nirS*. Arrows present the direction and magnitude of soil properties and lant associated with soil denitrifiers. GP1-H, GP4-H, GP7-H represent grazing prohibition for one, four, and seven years in high marsh, respectively; GP1-M, GP4-M, GP7-M represent grazing prohibition for one, four, and seven years in middle marsh, respectively.

**Figure 7:**
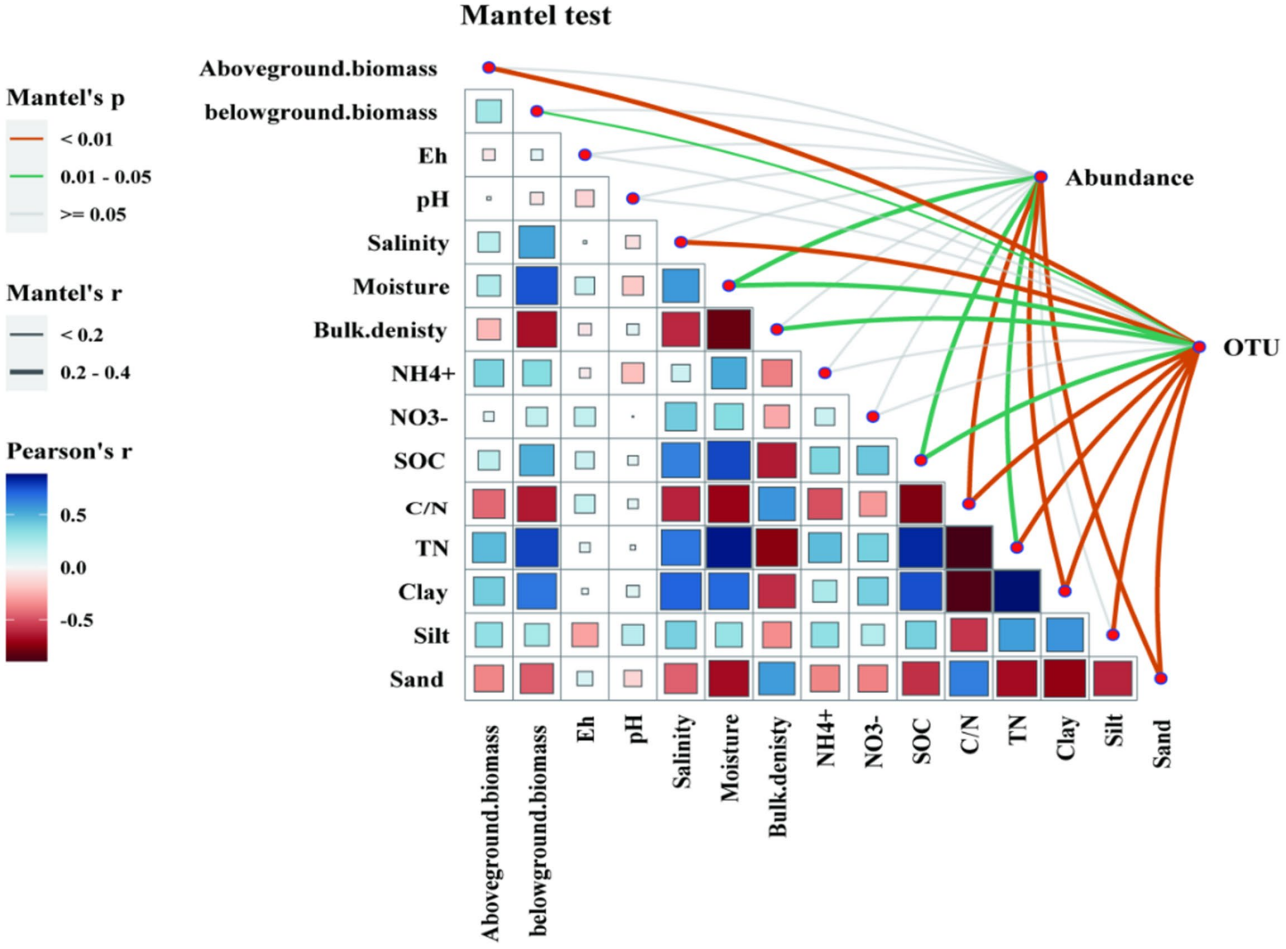
The results of Spearman’s correlation analysis and Mantel’s test. A Spearman’s rank correlation analysis of denitrifying gene abundance with soil properties and vegetation characteristics. The blue and red colors show, positive and negative relationships between variables, respectively. The deeper the color and the larger the square, the stronger correlation relationships. Significant correlations are indicated by *p < 0.05, **p < 0.01, ***p < 0.001. Functional microbial composition (based on OTU) was related to each environmental factor by partial Mantel’s test using Bray-Curtis distance shown in an interaction network in the top right corner of the Figure. Edge color corresponds to the Mantel’s R statistic for the corresponding distance correlations. Insignificant correlations (*p* > 0.05) are shown by grey lines in Mantel’s test.

RDA was conducted to analyze the relationship between the *nirK*- and *nirS*-type denitrifying communities and selected soil properties (SOC, C/N, bulk density, pH, NH_4_^+^, NO_3_^−^, salinity, moisture, Eh, clay, silt, and sand) and plant biomass (above-biomass and belowground). The RDA plot for different grazing prohibition treatments in the salt marshes showed that soil properties and plants explained 50.35% and 61.10% of the variation in the *nirK*-type and *nirS*-type denitrifying communities (Figures 5A,B), respectively. In general, the abundance of *nirK* was the most affected by SOC, salinity, aboveground biomass, and TN (Figures 6, 7; Supplementary Figure S1). Salinity, C/N, and SOC were the dominant predictors of variation in the *nirS*-type denitrifying communities (Figures 6B, 7 and Supplementary Figure S1).

## 4. Discussion

### 4.1. Effects of grazing prohibition on plant biomass and soil properties

As the duration of grazing prohibition increased, the above-and belowground biomass increased (Figures 2A,B), indicating a positive effect of grazing prohibition on productivity in salt marshes. This change was caused by increased competition and the exclusion of less competitive species during late successional stages (Cheng et al., 2016; Spangler et al., 2021). For example, *Phragmites australis* expanded significantly in the later stages (GP7), whereas species such as *Carex scabrifolia, Imperata cylindrica, Scirpus mariqueter, Scirpus validus*, declined (Table 1).

Grazing prohibition usually influences soil properties, and the observed effects often differ based on the grazing prohibition time and geographical environment (Song et al., 2019). In the present study, we analyzed the effects of grazing prohibition on the main soil physical and chemical properties that affect the abundance and diversity of denitrifiers, that is, soil pH, moisture, bulk density, SOC, NO_3_^−^ and NH_4_^+^ concentrations, and C/N (Pérez-Brandan et al., 2019). We found that the SOC content increased with grazing prohibition time in both the high and middle marshes. These findings are consistent with the previously published results for grasslands (Dong et al., 2021). For instance, Wang Z. et al. (2019) found that soil carbon storage increased after grazing exclusion in an overgrazed grassland, mainly because of the recovery of vegetation community along with grazing prohibition time and a higher return of C through aboveground litter, which further stimulated sediment accumulation and increased carbon content with vegetation growth (Belsky, 1992; Su et al., 2005; Liu et al., 2019; Dunn and Minderhoud, 2022).

The effects of grazing prohibition on soil N concentrations and forms in salt marshes have also been studied because *N* is the predominant limiting factor for plant primary productivity and microbial function during salt marsh restoration (Wang et al., 2012). In the present study, we observed that TN concentrations significantly increased with grazing prohibition time, whereas no significant effects of grazing prohibition on soil NO_3_^−^ or NH_4_^+^ were observed. These results are in line with those by Paul et al. (2013), who reported no differences in NO_3_^−^ and NH_4_^+^ between grazing prohibition treatments. Vegetation intercepts more inorganic nitrogen but also enhances the NH_4_^+^ absorption in the sediment along with grazing prohibition time (Berg et al., 1997; Martin and Reddy, 1997). Moreover, as the prohibition time increased, nutrients from cattle manure and urine entering the soil were gradually diluted, resulting in no differences in NO_3_^−^ and NH_4_^+^ (Li et al., 2022).

Salinity and bulk density showed a downward trend with grazing prohibition time, probably because of the recovery of plant communities and increasing above-and belowground biomass, which led to higher soil moisture and more plant litter in the sediments (Figures 2A,B,H). Similar results have been reported for grasslands (Chaneton and Lavado, 1996; Di Bella et al., 2014). No grazing prohibition time affected the soil pH, which is consistent with many previous studies (Yang Z. et al., 2017; Tang et al., 2020; Li et al., 2022).

### 4.2. Effects of grazing prohibition on the abundances of *nirS* and *nirK*

In this study, *nirS* abundance significantly increased with grazing prohibition time, whereas *nirK* abundance was not affected (Figure 3). Our results are consistent with the findings of Wang et al. (2021), who found that *nirS* abundance increased in grazing-prohibited grassland soils, and with those of Song et al. (2019) who reported the accumulation of denitrifying genes after grazing exclusion. Furthermore, we found that the *nirS* abundance was higher than that of *nirK* in each grazing prohibition phase (Figure 3), in accordance with Yin et al. (2015) and Yang et al. (2018). These results indicate that *nirS* genes are more sensitive to grazing prohibition and should be the first candidates to be used as an indicator of the microbial denitrification process during salt marsh restoration.

The positive correlations between *nirS* abundance and TN, SOC, salinity, silt, and clay (Supplementary Figure S2) indicate that a higher return of C and changes in the depositional environment after grazing prohibition could support high concentrations of substrates and anaerobic environments for denitrifying bacteria (Li et al., 2021). Similarly, indicators affecting soil oxygen concentrations, such as soil moisture, bulk density, and sand content, correlated with *nirK* abundance (Nishisaka et al., 2019). These results suggest that substrate and soil oxygen concentrations are the two main driving factors for *nirK*- and *nirS*-type microorganism activity in the process of salt marsh restoration during the grazing prohibition stage.

### 4.3. Effects of grazing prohibition on the diversity and structure of the bacterial communities of *nirS*- and *nirK* denitrifiers

The OTU richness, ACE, and Chao1 indices of *nirS* denitrifying bacterial communities were significantly higher than those of *nirK* denitrifying bacterial communities in salt marshes under different grazing prohibition treatments (Table 2). These results are similar to previous findings (Han et al., 2020), indicating that the *nirS*-denitrifying bacterial communities had higher community richness than *nirK*-denitrifying bacterial communities.

Soil denitrifying bacterial communities that result in the loss of nitrogen are largely affected by ecosystem management (Han et al., 2020), which alters the soil physicochemical properties (Kuramae et al., 2012; Xie et al., 2014). In the *nirK*-denitrifying bacterial communities, some species in the genera *Bradyrhizobium* and *Rhizobium* were significantly affected by grazing prohibition. Among *nirS*-denitrifying bacterial communities, *Sulfurifustis*, *Azoarcus*, and *Steroidobacter* were significantly affected by grazing prohibition. Wang Z. et al. (2019) reported that ecosystem restoration influenced the abundance of denitrifying bacteria, possibly because of changes in soil carbon availability due to grazing prohibition. The abundance of *nirK*- and *nirS*-denitrifiers varied according to grazing prohibition and correlated with SOC, TN, and salinity. Soil SOC and TN have previously been reported to serve as metabolic substrates that directly or indirectly influence denitrifying bacterial communities (Chen et al., 2020). Moreover, several studies have consistently found that the abundance of denitrifying bacterial communities were strongly influenced by salinity (Franklin et al., 2017; Fu et al., 2019; Pan et al., 2023). We found that salinity decreased along with grazing prohibition times (Figure 2E), which may result the changes in *nirS*- and *nirK*-types denitrifying bacterial communities (Zaghmouri et al., 2018). Therefore, the composition of the *nirS*- and *nirK*-denitrifying bacterial communities in the soil varied significantly among the grazing prohibition treatments, largely due to changes in soil properties in salt marshes.

## 5. Conclusion

In summary, our results demonstrate changes in *nirS*- and *nirK*-denitrifying bacterial communities with grazing prohibition time in salt marshes. The *nirS* abundance increased with grazing prohibition time, and was higher than *nirK* abundance in all treatments. The OTU richness, ACE, and Chao1 indices of the *nirS*-denitrifying bacterial communities were significantly higher than those of the *nirK*-denitrifying bacterial communities. Grazing prohibition significantly altered the abundance, OTU richness, ACE, and Chao1 indices of the *nirS*-denitrifying bacterial communities, whereas it only minimally affected the structure of the *nirK*-denitrifying bacterial community. In addition, shifts in the abundance, diversity, and structure of the *nirS* and *nirK*-denitrifying bacterial communities were associated with variations in soil properties, especially SOC, TN, and salinity. Our results provide insights into the diverse responses of *nirS*- and *nirK*-denitrifying bacterial communities to grazing prohibition in salt marshes.

## Data availability statement

The original contributions presented in the study are included in the article/Supplementary material, further inquiries can be directed to the corresponding authors.

## Author contributions

JW and NL designed the study. NL conducted the work and analyzed the data. NL, JL, MN, MW, and JW wrote the manuscript. All authors contributed to the article and approved the submitted version.

## Funding

This work was funded by the National Natural Science Foundation of China (grant no. 31570513), Cooperation of Zhejiang Province and the Chinese Academy of Forestry (grant nos. 2023SY11 and 2021SY03), Zhejiang Province Commonwealth Projects (grant no. LQ23C030003), and the Foundation of Research Institute of Subtropical Forestry, Chinese Academy of Forestry (RISFZ-2023-01).

## Conflict of interest

The authors declare that the research was conducted in the absence of any commercial or financial relationships that could be construed as a potential conflict of interest.

## Publisher’s note

All claims expressed in this article are solely those of the authors and do not necessarily represent those of their affiliated organizations, or those of the publisher, the editors and the reviewers. Any product that may be evaluated in this article, or claim that may be made by its manufacturer, is not guaranteed or endorsed by the publisher.

## Abbreviations

ACE: abundance-based coverage estimator
ANOSIM: analysis of similarities
ANOVA: analysis of variance
nir: nitrite reductase
NMDS: non-metric multidimensional scaling
Nor: non-ripening
OTU: operational taxonomic unit
qPCR: quantitative polymerase chain reaction
RDA: redundancy analyses
SOC: soil organic carbon
SE: standard error
SoC: substances of concern
TN: total nitrogen
