# Supplementary material for: Effects of grazing prohibition on nirK- and nirS-type denitrifier communities in salt marshes
Source: Front Microbiol. 2023 Jul 26;14:1233352. doi: 10.3389/fmicb.2023.1233352 (PMC10411955; doi:10.3389/fmicb.2023.1233352)
Supplement: Supplementary file 1 [file Table_1.DOCX]

**Table S1** Results from the general linear models (GLM) showing the importance of grazing prohibition time and marsh zone from *nirK* and *nirS* gene copy numbers

| Factor | *nirK* | *nirS* |
| --- | --- | --- |
| Marsh zone | < 0.01 | 0.132 |
| Grazing prohibition time | 0.534 | < 0.01 |
| Grazing prohibition time × Marsh zone^a^ | 0.053 | 0.892 |

^a^ Interaction of grazing prohibition time and marsh zone.
