# Supplementary material for: Effects of grazing prohibition on nirK- and nirS-type denitrifier communities in salt marshes
Source: Front Microbiol. 2023 Jul 26;14:1233352. doi: 10.3389/fmicb.2023.1233352 (PMC10411955; doi:10.3389/fmicb.2023.1233352)
Supplement: Supplementary file 2 [file Table_2.DOCX]

**Table S2 Gr**azing prohibition time and marsh zone and their interaction on alpha diversity of nirK and nirS gene. P-values are bold-typed at p ≤ 0.05.

| Genes | Alpha diversity indices | Grazing prohibition time | | Marsh zone | | Grazing prohibitiontime×Marsh zone | |
| --- | --- | --- | --- | --- | --- | --- | --- |
|  |  | F | p-value | F | p-value | F | p-value |
| *nirK* | OTU richenss | 7.586 | **0.011** | 3.245 | 0.057 | 6.713 | **0.005** |
|  | ACE | 7.691 | **0.011** | 3.064 | 0.065 | 7.116 | **0.004** |
|  | Chao1 | 7.864 | **0.010** | 2.748 | 0.084 | 7.373 | **0.003** |
|  | Simpson | 0.938 | 0.342 | 1.966 | 0.162 | 0.966 | 0.395 |
|  | Shannon | 0.685 | 0.416 | 5.107 | **0.014** | 1.638 | 0.215 |
| *nirS* | OTU richenss | 30.686 | **0.000** | 6.731 | **0.005** | 22.231 | **0.000** |
|  | ACE | 12.757 | **0.002** | 4.325 | **0.025** | 21.198 | **0.000** |
|  | Chao1 | 10.073 | **0.004** | 3.322 | 0.053 | 18.216 | **0.000** |
|  | Simpson | 25.693 | **0.000** | 0.454 | 0.641 | 3.701 | **0.040** |
|  | Shannon | 65.016 | **0.000** | 1.331 | 0.283 | 1.698 | 0.204 |
