# Supplementary material for: Effects of grazing prohibition on nirK- and nirS-type denitrifier communities in salt marshes
Source: Front Microbiol. 2023 Jul 26;14:1233352. doi: 10.3389/fmicb.2023.1233352 (PMC10411955; doi:10.3389/fmicb.2023.1233352)
Supplement: Supplementary file 3 [file Data_Sheet_1.docx]

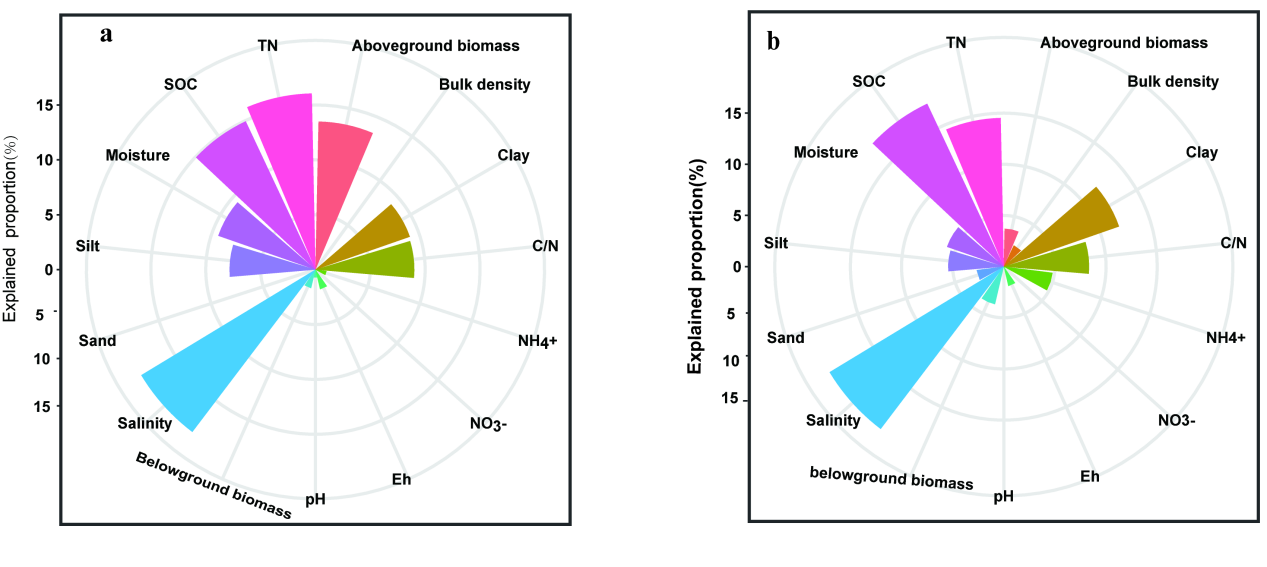


**Fig.S1.** Relatuve importance of plant and soil properties as predictors of denitrifying communities. (a) nirK, (b) nirS.
