# Supplementary material for: Effects of grazing prohibition on nirK- and nirS-type denitrifier communities in salt marshes
Source: Front Microbiol. 2023 Jul 26;14:1233352. doi: 10.3389/fmicb.2023.1233352 (PMC10411955; doi:10.3389/fmicb.2023.1233352)
Supplement: Supplementary file 4 [file Data_Sheet_2.docx]

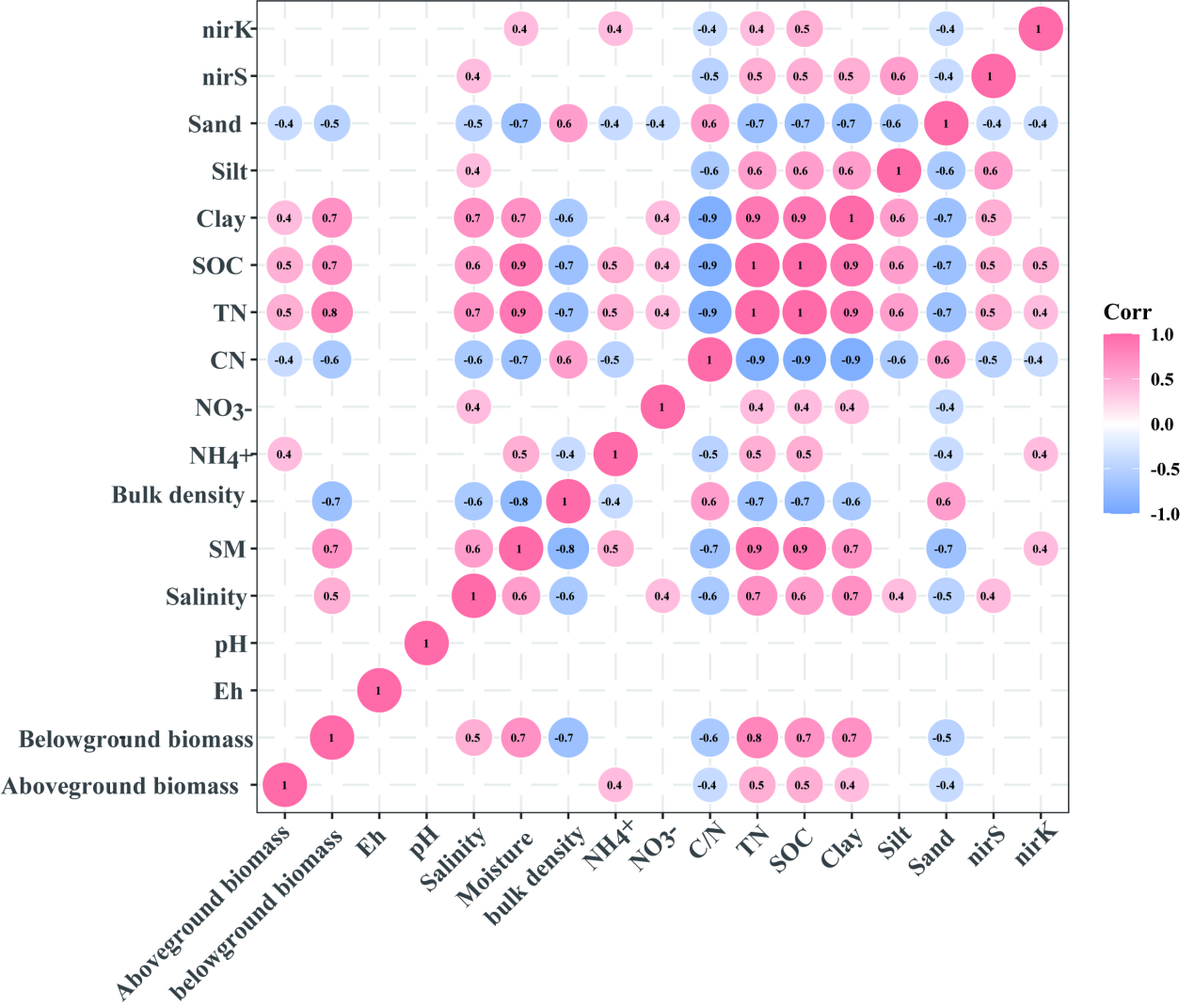


**Fig.S2.** A Spearman's rank correlation analysis of denitrifying gene abundance with soil properties and vegetation characteristics. The blue and red colors show, respectively, positive and negative relationships between variables. The deeper the color and the larger the square, the stronger correlation relationships. The value in the circle indicates the correlation data.
